# Patient and physician views of shared decision making in cancer

**DOI:** 10.1111/hex.12564

**Published:** 2017-05-02

**Authors:** Nina P. Tamirisa, James S. Goodwin, Arti Kandalam, Suzanne K. Linder, Susan Weller, Stella Turrubiate, Colleen Silva, Taylor S. Riall

**Affiliations:** ^1^ Department of Surgery The University of Texas Medical Branch Galveston TX USA; ^2^ The University of California, San Francisco‐East Bay Oakland CA USA; ^3^ Sealy Center on Aging The University of Texas Medical Branch Galveston TX USA; ^4^ Department of Family Medicine The University of Texas Medical Branch Galveston TX USA; ^5^ Department of Oncology The University of Texas Medical Branch Galveston TX USA

**Keywords:** cancer, communication, patient preference, patient‐centered outcomes research, treatment

## Abstract

**Context:**

Engaging patients in shared decision making involves patient knowledge of treatment options and physician elicitation of patient preferences.

**Objective:**

Our aim was to explore patient and physician perceptions of shared decision making in clinical encounters for cancer care.

**Design:**

Patients and physicians were asked open‐ended questions regarding their perceptions of shared decision making throughout their cancer care. Transcripts of interviews were coded and analysed for shared decision‐making themes.

**Setting and participants:**

At an academic medical centre, 20 cancer patients with a range of cancer diagnoses, stages of cancer and time from diagnosis, and eight physicians involved in cancer care were individually interviewed.

**Discussion and conclusions:**

Most physicians reported providing patients with written information. However, most patients reported that written information was too detailed and felt that the physicians did not assess the level of information they wished to receive. Most patients wanted to play an active role in the treatment decision, but also wanted the physician's recommendation, such as what their physician would choose for him/herself or a family member in a similar situation. While physicians stated that they incorporated patient autonomy in decision making, most provided data without making treatment recommendations in the format preferred by most patients. We identified several communication gaps in cancer care. While patients want to be involved in the decision‐making process, they also want physicians to provide evidence‐based recommendations in the context of their individual preferences. However, physicians often are reluctant to provide a recommendation that will bias the patient.

## 1 INTRODUCTION

Shared decision making in the clinical encounter is the process by which patients, physicians and caregivers arrive at treatment decisions together based on clinical evidence within the context of a patient's personal preferences. This is an increasingly important concept in the practice of medicine and has been included as a provision in the Affordable Care Act.^1^ Several studies have evaluated the shared decision‐making model and developed a framework through which the essential concepts can be operationalized in clinical practice.^2^, ^3^ The shared decision‐making process depends on the physician's ability to communicate the benefits and risks of various treatment options and to clarify patient values and elicit preferences. This discussion is particularly important in cancer care where patients are provided with several options that often involve trade‐offs between quantity and quality of life.^4^

The benefits of shared decision making include improved patient satisfaction, an increased likelihood of adherence to treatment, and a reduction in health‐care costs.^1^, ^5^, ^6^ Despite these benefits, several studies have shown that shared decision making is not commonly achieved in clinical practice. In a systematic review, Gaston et al.^7^ reported widespread misunderstanding of prognosis and extent of disease in patients with cancer, mostly due to physician reluctance to openly discuss a poor prognosis. In interview studies, cancer patients’ preferences for an active, collaborative or passive role varied widely,^9^, ^10^ and physicians’ perceptions of the patient's desired role were inconsistent with actual patient preferences.^8^, ^9^

The aim of the current study was to explore patient and physician perceptions of shared decision making in clinical encounters for cancer care at one medical centre. We hope to lay the groundwork for larger, multicenter studies, leading to interventions to improve shared decision making in this patient population.

## 2 METHODS

The study involved direct communication with physicians involved in cancer care and patients with a recent diagnosis of cancer. The Institutional Review Board at the University of Texas Medical Branch approved the study and waived written consent due to minimal risk to study participants. Verbal consent was obtained from each participant, and a compensation of a $25 gift card was offered to patients for their participation.

### 2.1 Participants and recruitment

We interviewed 20 patients with cancer in one‐on‐one interviews. The types of cancers included breast cancer, pancreatic cancer, cervical cancer, endometrial cancer and melanoma. Fully 50% of patients were diagnosed with metastatic disease and 50% with non‐metastatic disease. Patients were asked about participation in this study by their physician or clinical oncology nurse. Patients who expressed interest in participating were referred to our study personnel to schedule an interview. When possible, interviews were completed at the time of the clinical encounter. When this was not possible, study coordinators arranged interviews to coincide with future visits or at a setting of the patient's choice. A purposive sampling of eight physicians, including surgeons, oncologists, gastroenterologists and palliative care physicians, were interviewed. All physicians on a tumour board email mailing list at the University of Texas at Galveston were individually emailed. The goals and design of the study were explained, and physicians were asked to participate. Those physicians who agreed to participate were subsequently verbally consented and interviewed.

### 2.2 Data collection

All interviews were done by one individual, a physician (NPT). Separate and standard interview guides with probes for clarification from patients and physicians were used in semi‐structured interviews. The interview guides were pilot tested with four participants for feasibility. Patients were asked open‐ended questions regarding their perceptions of shared decision making throughout their cancer care. Treatment expectations were elicited using questions adapted from the Cancer Care Outcomes Research and Surveillance patient survey.^11^ Interview topics focused on the presentation of treatment options, information on the benefits and risks of each option, elicitation of patient preferences, presentation of the physician's recommendation and current strategies to improve shared decision making. Physicians were also asked open‐ended questions regarding their practice patterns and approach to discussing the diagnosis as well as the treatment options with their patients. The interview topics were the same as those asked of patients, focusing on how treatment options are presented, delivery of information, how benefits and risks are communicated, and how they make recommendations to patients while respecting patient autonomy.

In addition, using a free‐listing technique, patients were asked to list the qualities most important to promote shared decision making in a treating physician. This technique is designed to establish domains and identify what is most culturally relevant to participants. The number of patients who mentioned specific qualities was tallied. Interviews were approximately 45‐60 minutes in length. All interviews were audio‐recorded and transcribed verbatim.

### 2.3 Data analysis

A data immersion technique was used initially, whereby original transcripts were reviewed without coding to identify emerging themes. The development and application of the code structure was performed by a single investigator (NPT) with subsequent review by a second investigator (SKL).^12^ Grounded theory was used for reviewing transcripts whereby codes were inductively assigned to emerging themes allowing for a clear and comprehensive code structure. Codes were compared across transcripts to ensure that the context was similar within each code. To establish intercoder reliability, codes were assigned by the two coders to transcripts that were not included in this study. With approximately 80% agreement, the remainder of the coding was completed independently (NPT). Codes were used to define themes, and subcodes were used to generate domains within themes: for example, classifying participant perspective about a theme as positive, negative or indifferent. The code structure was finalized at the point of saturation when no new themes were identified. Transcripts of interviews were coded and analysed using NVivo 10 (QSR International, Melbourne, Australia). The number of patients and physicians who mentioned specific themes was tallied.

## 3 RESULTS

We interviewed twenty patients with cancer (10 men and 10 women) and eight physicians. The mean age for our patient cohort was 63.3±9 years. All patients were interviewed within 6 months of diagnosis (median 4 months, range 3‐6 months). The patients included 17 females with breast cancer (12), pancreatic cancer (1), lymphoma (2), cervical cancer (1) and melanoma (1), and three males with lymphoma, pancreatic cancer and colon cancer. All patients received chemotherapy, 87.5% underwent surgery and 75.0% underwent radiation. The eight physicians included in this study were four surgeons, two oncologists, one palliative care physician and one gastroenterologist, with a wide range in the number of years in practice (median 15.3 years, range 8 months to 33 years).

### 3.1 Shared decision‐making themes

Four major themes that emerged as factors influencing shared decision making from patient and physician perspectives were as follows: (i) information provided, (ii) patient autonomy, (iii) communication of patient and physician priorities and (iv) physician's recommendation. Table 1 reports examples of these themes with verbatim quotations from patients and physicians.

**Table 1:** Examples of themes across components of shared decision making

Patients	Physicians
*Information too detailed*	*Information overwhelms patient*
“Things in writing always help. Too much paper gets to be a whole lot though.” ‐75 y/o female pancreatic casncer	“I don't want to give them a lot of information up front and overwhelm them with a lot of data.” ‐Oncologist
“They hand you the whole thing that has every single slide and I think I'm going to go back and read this and go over it but I never do.” ‐79 y/o female non hodgkin's lymphoma	“We have lots of stuff… but I don't know that they read it and I do think that it's overwhelming.” ‐Surgeon
*Information inadequate*	*Information easy to understand*
“No one ever asked me what my background was so they could know what level of information I could receive.” ‐58 y/o Male colon cancer	“We have lots of written information; it's usually well understood by people with a high school diploma.” ‐Surgeon
“It was one of those things where I had to trust doctors and my gut and my heart, but I don't feel like I've been an educated patient.” ‐42 y/o female melanoma	“We have lots of stuff from the American Cancer Society that we give them with all the information they need about chemotherapy and radiation.” ‐Surgeon
*Patient autonomy*	
“I would prefer to know that I am making the decision. I mean after all it is your body right?” *‐*65 y/o female breast cancer	“I think it's most of the time their decision. You give them the data but in most cases, it's their decision.” ‐Oncologist
“It was my decision. My husband was there, my son was there, and it was up to me… I decided.” ‐85 y/o female pancreatic cancer	“I think that the patients need to have the final say because we may think we are doing the best for them but that may not be the case.” ‐Gastroenterologist
*Communication of patient and physician priorities*	
“You ought to be talked to about your self‐image, cancer changes your whole self‐image.” ‐64 y/o female breast CA	“If the choice is, you are going to have to wear a wig for a while but you are going to be around, I am much more into doing that” ‐Oncologist
“Not having the option was an option but not really, you know, everyone advised the chemo so I knew I had to have the chemo.” ‐75 y/o female pancreatic CA	“It's frustrating when you know what the right thing to do is but try to convince patients to see otherwise, there is a gap in knowledge between patients and physicians.” ‐Surgeon
*Physician recommendation*	
“My doctor told me that I'm not going to come out of this unscathed, he convinced me that this was what was needed by saying if this was my mom or sister I would tell them to get it done.” ‐59 y/o female breast CA	“I always tell them that the standard of care that I am able to offer you may not be something you want. I think you should go and get a second opinion.” ‐Surgeon
“Many people get frustrated when a doctor won't tell them what they recommend. And that is very hard for patients.” ‐69 y/o female breast CA	“I tried to put in my bias and make a recommendation with a patient a long time ago and the patient didn't like it.” ‐Gastroenterologist
“Sometimes, you have to put it on the line. “What do you think I ought to do?” I think a good physician will make a recommendation at that point. The patient deserves that and I think that in medicine, we have maybe gone a little far the other way now in the patient involvement.” ‐66 y/o female breast CA	“I always try to stay really objective, it's hard to take your opinion completely out of the decision making, but I do it by following the data.” ‐Oncologist

### 3.2 Information

All physicians reported that they provided information regarding treatment options, risks and benefits. One theme shared by patients and physicians was that the information could be overwhelming. Patients frequently mentioned that the written information provided was too detailed and that the physicians did not assess the level of information they were able or wished to receive. Examples of these themes are reported in Table 1.

### 3.3 Patient autonomy, and communication of patient and physician priorities

Most patients wanted to play an active role in the treatment decision. For example, a 65‐year‐old woman with breast cancer stated, “I would prefer to know that I am making a decision. I mean, after all it is your body, right?” In addition, most physicians were aligned with an active patient role and mentioned incorporating patient autonomy into the final treatment decision; for example, “I think patients need to have the final say because we think we are doing the best for them, but that may not be the case.” At the same time, a prevalent theme among physicians was ambivalence about the shared decision‐making processes and patient autonomy. For example, a surgeon stated: “It's frustrating when you know what the right thing to do is but try to convince the patients to see otherwise …” (Table 1).

### 3.4 Physician recommendation

Most patients wanted the physician's treatment recommendation, including knowing what the physician would choose for him/herself or a family member in a similar situation; for example, “many people get frustrated when a doctor won't tell them what they recommend” (Table 1). Some physicians indicated that they gave treatment recommendations, but most were negative or ambivalent; for example, “I tried to put in my bias and make a recommendation with a patient a long time ago and the patient didn't like it” (Table 1).

### 3.5 Patient and physician perceptions of the shared decision‐making process

Patients felt that physician behaviour and communication were major factors that facilitated shared decision making in the clinical encounter. This was found in the semi‐structured interview questions. For example, a woman with breast cancer stated, “I have had that physician that comes in and they make no contact with me. They go right to the computer and start charting or looking up the report and well then, we are all just robots. The doctor should sit and talk to you and listen and ask some pointed questions.” We also assessed this more directly using a free‐listing technique, asking each patient all the qualities in a physician that would promote shared decision making. The most important qualities in physicians who were perceived as incorporating patient preferences were physicians who sat down, explained treatment options verbally, spoke while looking at the patient instead of at the computer and took the time to ensure that all questions were answered (Table 2).

**Table 2:** Free listing of patient‐reported qualities of physicians who exhibit shared decision making

Qualities	N=20
Someone who shows compassion, commitment	20
Does not sit behind a laptop	
Sits down	
Talks eye to eye	
Empathetic	
Spends time with you	20
Answers all questions	
Reads my chart before they come in	
Not seeming like they have to rush	
Ability to listen	17
Hears what I am saying and takes it into account	
Tried to accommodate me	
Knowledgeable	15
Prints out the latest information	
Has the right answers	
Says what the plan is	2
Gives me an explanation	
Honesty	2
Does not sugar coat anything	

We asked physicians how to improve shared decision making in clinical practice. A common theme involved reinforcing patient knowledge through multiple visits and repetition of information: “It requires a lot of visits for the patient to come back and have a clear understanding of what they are diving into” (Surgeon). In addition, all physicians mentioned using the multidisciplinary tumour board to expedite care and ensure that patients are given a plan agreed upon by all participating physicians: “I always tell the patients that we are going to be discussing your case in the tumor board; several of my colleagues are going to be there; and we are going to talk about you and come up with a plan” (Oncologist). One physician mentioned discussing patient preferences during tumour board presentations.

## 4 DISCUSSION

We identified concerns about the shared decision‐making process for cancer patients with regard to both delivery of the information necessary to make an informed decision and elicitation of patient preferences. Most patients reported that physicians did not adequately assess or meet their informational needs. Shared decision making occurs when patients and physicians arrive at treatment decisions together, based on the best available evidence and weighted according to an individual patient's values and preferences. Without understanding their treatment options and prognosis, patients cannot truly make a decision based on their personal preferences.

Previous studies have demonstrated that, for patients with cancer, being a part of the decision‐making process meant being provided with adequate information at various stages of treatment.^4^, ^13^, ^14^, ^15^ However, the level of information desired varies among patients.^16^, ^17^, ^18^, ^19^, ^20^ In a study using in‐depth interviews of 17 patients with cancer, all patients wanted basic information on treatment, but only a third of patients wanted as much information as possible.^16^ In our study, some patients felt their physicians did not assess the level of information they were able or wished to receive, and most mentioned that information provided was not helpful. In contrast, most physicians we interviewed perceived that the information they provided to patients was sufficient and easy to understand.

National Comprehensive Cancer Network Guidelines recommend multidisciplinary consultation regarding complex cancer treatment.^21^ After consensus is reached, patients are often told the best options as per the tumour board recommendations. We found that, while all of the physicians mentioned using the tumour board to coordinate and expedite care, only one physician mentioned incorporating patient preference into discussions at these conferences.

Providing a treatment recommendation in concordance with a patient's preferences is a central tenet in the shared decision‐making process. Previous studies have demonstrated that physicians do not always provide a recommendation on which of the treatment options they think is preferable.^22^ When providing a recommendation, many physicians do not disclose their personal opinions on the optimal treatment and instead focus on providing information on the risks and benefits of each option, leaving the choice to the patient.^23^, ^24^, ^25^ In our study, most patients spontaneously mentioned that they preferred physicians to provide recommendations by addressing questions like “What would you do if this were your relative?” or “What would you do if you were in my situation?”

It is not surprising that many patients perceive serious problems in shared decision making. The model of medical decision making has changed dramatically in a generation, from authoritarian to shared decision making. This means that many physicians and patients “grew up” under the old model and experience different levels of comfort adapting to the new one. Nor is it clear that this evolution in models of decision making is now stable, or whether it will continue to rapidly change. Given the dynamic nature of models of medical care, it is important to continually assess patient preferences and perceptions, along with the views of the treating physicians. This is best accomplished by including a qualitative component, to allow for the full range of responses.

Increased funding for patient‐centred outcomes research is a major provision of the Affordable Care Act.^26^, ^27^ Interventions to improve shared decision making have resulted in increased patient knowledge and a greater likelihood of receiving care aligned with patient preferences.^1^, ^5^, ^6^, ^7^, ^27^, ^28^ Our study identifies continuing gaps in knowledge and assessment of the informational needs of patients as key barriers to shared decision making. These can serve as targets for interventions to improve patient satisfaction and clinical outcomes.

There are several limitations to this study. First, the sample of participants limits generalization to all patients and physicians involved in cancer care. Patients included in this study varied by diagnosis, stage and time from initiation of treatment. The physician sample was small, including physicians at a single centre. A more representative sample of physicians or samples of physicians from several regions could potentially produce different results than what we obtained. As a pilot study, the goal of this project was to identify the barriers and facilitators to shared decision making in cancer care for all patients with a diagnosis of cancer and physicians involved in cancer care. Some of these barriers seem relatively easily addressable, such as asking patients whether they want a recommendation about treatment and then providing one if asked. Others, such as the failure of written materials to meet the patient's needs for information, have seemingly not improved despite considerable effort by many organizations in developing such materials.

Cancer care is complex, and patients are faced with several treatment decisions that impact quality and quantity of life. Shared decision making in cancer care highlights the need for ensuring that patients understand the nature of their disease and have adequate knowledge of treatment risks, benefits and outcomes. Communication of information is the foundation for providing recommendations to patients in the context of their clinical characteristics and personal preferences. In our pilot study, we identified several communication gaps in cancer care. Better physician assessment of patients’ informational needs and development of their ability to elicit patients’ preferences will improve the shared decision‐making process in cancer patients.

## CONFLICT OF INTERESTS

None.
